# Novel Lipid Nanoparticles Stable and Efficient for mRNA Transfection to Antigen-Presenting Cells

**DOI:** 10.3390/ijms25031388

**Published:** 2024-01-23

**Authors:** Kang Chan Choi, Do Hyun Lee, Ji Won Lee, Jin Suk Lee, Yeon Kyung Lee, Moon Jung Choi, Hwa Yeon Jeong, Min Woo Kim, Chang-Gun Lee, Yong Serk Park

**Affiliations:** 1 Department of Biomedical Laboratory Science, Yonsei University, Wonju 26493, Republic of Korea; cellbiologylab@yonsei.ac.kr (K.C.C.); dlehgus499@gmail.com (D.H.L.); jiwon4004@yonsei.ac.kr (J.W.L.); yunni8011@naver.com (Y.K.L.); cglee@yonsei.ac.kr (C.-G.L.); 2 Regeneration Medicine Research Center, Yonsei University Wonju College of Medicine, Wonju 26426, Republic of Korea; cherry0613@yonsei.ac.kr; 3 Division of Hematology/Oncology, Brown University and Rhode Island Hospital, Providence, RI 02903, USA; cmj4458@gmail.com; 4 Department of Biotechnology, College of Life Sciences and Biotechnology, Korea University, Seoul 02841, Republic of Korea; cca1987@korea.ac.kr; 5 Division of Breast Surgery, Department of Surgery, Yonsei University College of Medicine, Seoul 03722, Republic of Korea; minwookim@yuhs.ac

**Keywords:** mRNA delivery, lipid nanoparticles, drug delivery system

## Abstract

mRNA vaccines have emerged as a pivotal tool in combating COVID-19, offering an advanced approach to immunization. A key challenge with these vaccines is their need for extremely-low-temperature storage, which affects their stability and shelf life. Our research addresses this issue by enhancing the stability of mRNA vaccines through a novel cationic lipid, O,O′-dimyristyl-N-lysyl aspartate (DMKD). DMKD effectively binds with mRNA, improving vaccine stability. We also integrated phosphatidylserine (PS) into the formulation to boost immune response by promoting the uptake of these nanoparticles by immune cells. Our findings reveal that DMKD-PS nanoparticles maintain structural integrity under long-term refrigeration and effectively protect mRNA. When tested, these nanoparticles containing green fluorescent protein (GFP) mRNA outperformed other commercial lipid nanoparticles in protein expression, both in immune cells (RAW 264.7 mouse macrophage) and non-immune cells (CT26 mouse colorectal carcinoma cells). Importantly, in vivo studies show that DMKD-PS nanoparticles are safely eliminated from the body within 48 h. The results suggest that DMKD-PS nanoparticles present a promising alternative for mRNA vaccine delivery, enhancing both the stability and effectiveness of these vaccines.

## 1. Introduction

In recent years, the delivery of antigenic mRNAs into the cytoplasm and their translation into antigenic proteins to acquire immunity has been well documented [1,2,3]. mRNA molecules exhibit low immunogenicity, allowing for repeated administration, and their single-stranded nucleic acid structure is beneficial for large-scale production [4,5,6]. Additionally, as mRNA metabolism is restricted to the cytoplasm, the risk of specific sequence integration into the genome is minimal, rendering them safer than DNA vaccines [7,8]. However, mRNA’s inherent structural vulnerability, particularly its susceptibility to RNase degradation, can lead to inefficient delivery, consequently reducing its therapeutic effectiveness [9,10,11,12].

Various carriers have been developed to address these structural challenges [13,14,15,16,17], among which lipid nanoparticles (LNPs) have been successful [18,19]. LNPs are typically composed of ionizable lipids, PEGylated lipids, cholesterol, and phospholipids. Ionizable lipids, which are neutral at physiological pH, are ionized under specific acidic conditions within the cell [20], facilitating endosomal escape and lysosomal avoidance [21,22,23,24]. PEGylated lipids, which are a combination of lipids and polyethylene glycol (PEG), enhance particle stability and prolong systemic circulation [25]. Similarly, cholesterol and phospholipids contribute to the structural integrity of the particles [26].

Despite these advancements, LNPs used for mRNA delivery require very inconvenient storage at −80 °C, reflecting a limitation in structural stability, especially when compared to the 4 °C storage requirement for DNA vaccines [27,28]. To overcome this shortcoming, mRNA was encapsulated in LNPs formulated with positively charged O,O′-dimyristyl-N-lysyl aspartate (DMKD) in this study.

DMKD is a positively charged lipid at physiological pH. This characteristic enables the efficient capture of negatively charged mRNA and maintains nucleic acid integrity even under harsh conditions during extended storage. In addition, a certain amount of phosphatidylserine (PS) is present in the particles to elicit an immune response. PS is located on the inner leaflets of healthy cell membranes. Its exposure on the cell surface attracts a variety of immune cells, including antigen-presenting cells, because it serves as an “eat me” signal to immune cells [29]. In this study, LNPs containing PS were prepared for mRNA delivery, particularly to antigen-presenting cells, including macrophages. Optimized and conditioned DMKD-PS nanoparticles for the stable and efficient delivery of mRNA molecules were compared with commercially available mRNA vaccination systems in terms of antigenic protein expression, in vitro cytotoxicity, and in vivo biodistribution in mice.

## 2. Results and Discussion

### 2.1. Preparation and Characterization of DMKD-PS

Previously, DMKD liposomes as novel and efficient pDNA-transfer vehicles were reported [30,31]. Conceptual illustrations of the DMKD-PS LNPs used in this study are presented in Figure S1. LNPs were prepared by dissolving each component in a 2:1 (*v*/*v*) chloroform: methanol solvent, followed by the lipid film method. Ten LNPs with various compositions were prepared. When the mole percentages of DMKD, cholesterol, and DSPE-PEG_2000_-amine were fixed at 48:40:4, respectively, the proportion of PS increased from 1 to 10 mol%. The lipid ratio that formed stable particles was then optimized using dynamic light scattering (DLS) analysis. Most DMKD-PS LNPs containing 1–10 mol% PS exhibited a homogeneous size distribution ranging from 100 to 200 nm, with no evidence of aggregation (Figure S2).

### 2.2. Optimization and Analyses of DMKD-PS Complexed with mRNA

**Figure 1:**
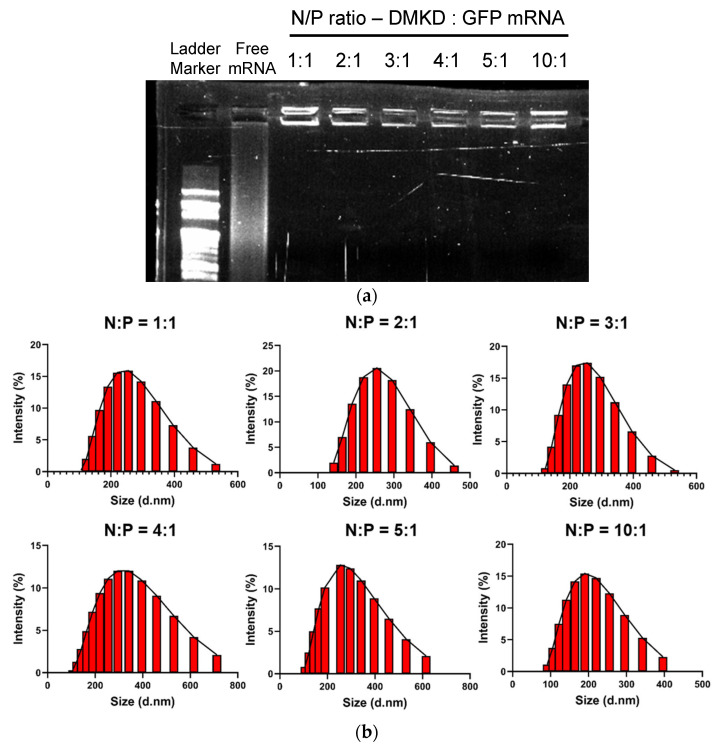
Gel retardation and sizing of DMKD-PS LNPs containing GFP mRNA prepared at various N/P ratios. (**a**) The mixtures of DMKD-PS:GFP mRNA at the indicated N/P ratios were run on 1% agarose gel and visualized by UV illumination. (**b**) The same samples were analyzed using DLS, and the results were presented as histograms.

The gel retardation assay indicated that most mRNA were retained in the wells of the agarose gel across all tested N/P ratios, suggesting that most mRNA were captured by DMKD-based LNPs (Figure 1a). Furthermore, DLS analysis of mRNA-complexed DMKD LNPs at various N/P ratios demonstrated a homogenous particle distribution without any aggregation (Figure 1b). To optimize the PS molar and N/P ratios to form stable and homogeneous particles, 40 different lipoplexes of DMKD-PS LNPs were prepared. These were derived from 10 types of PS, with molar ratios ranging from 1 to 10 mol% combined with four different N/P ratios (1:1, 2:1, 3:1, and 4:1). Dynamic light scattering (DLS) analysis revealed various polymorphic changes in the particles. At the 1:1 N/P ratio, some compositions did not form stable LNPs (Figure S3a). At a 2:1 N/P ratio, LNPs containing 1, 2, or 9 mol% PS demonstrated a homogenous particle distribution; however, small amounts of aggregates were also observed (Figure S3b). At a 3:1 ratio, the DMKD-PS with 2 mol% PS exhibited the most homogeneous and stable particle distribution (Figure S3c). However, all compositions with a 4:1 N/P ratio showed significant aggregation (Figure S3d). The most stable composition with the lowest polydispersity index (PDI) and no aggregation was determined to be the DMKD-PS LNPs, containing 2 mol% PS at a 3:1 N/P ratio. Therefore, all subsequent experiments were conducted using this formulation.

### 2.3. Physicochemical Properties of DMKD-PS

**Figure 2:**
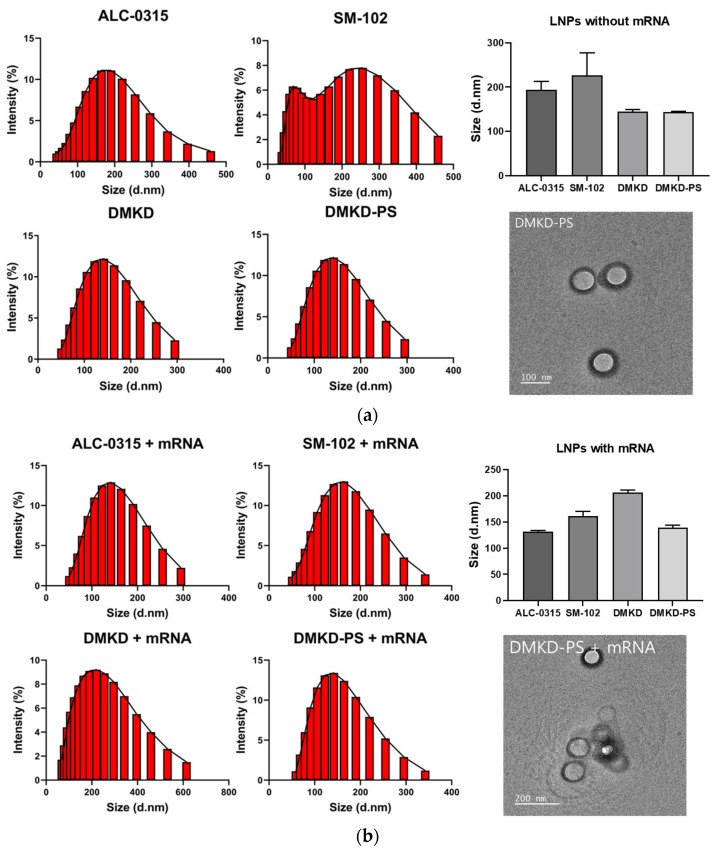
The size distribution of various LNPs. The size distributions of ALC-0315, SM-102, DMKD, and DMKD-PS LNPs without (**a**) and with (**b**) mRNA complexation were measured using a particle analyzer and are depicted in histograms, bar charts, and TEM images. Each error bar represents the mean ± S.D. from three separate experiments.

The physical properties of DMKD-PS and other LNPs (ALC-0315, SM-102, and DMKD) were analyzed before and after mRNA complexation. Particle size, zeta potential, and polydispersity indexes (PDIs) were measured using a particle analyzer (Figure 2) (Table 1). All LNP formulations were hydrated in normal saline and their final pH was adjusted to a physiological pH of 7.4.

Without mRNA complexation, SM-102 LNPs displayed a nonhomogeneous particle size distribution. The ALC-0315 and SM-102 LNPs had an average size of approximately 200 nm, whereas the DMKD and DMKD-PS LNPs measured 150 nm or less (Figure 2a). Upon mRNA complexation, a shift towards a more homogeneous size distribution was observed in all LNP types, accompanied by slight size changes (Figure 2b). In summary, the ALC-0315 and SM-102 LNPs exhibited reduced size and stabilized PDI values when mRNA was encapsulated. Although the DMKD and DMKD-PS LNPs increased in size, they remained below 200 nm. Notably, the DMKD LNPs showed a minor increase in PDI, and a decrease in zeta potential was observed for all LNPs after mRNA complexation (Table 1). The size distribution of the ALC-0315 and SM-102 lipid nanoparticles (LNPs) was found to be more stable when mRNA was encapsulated. These LNPs typically use an ionizable lipid forming a monolayer, with mRNA and the lipid creating a reverse micelle core for particle stabilization [32,33,34]. The same pattern was observed in LNPs using DMKD and DMKD-PS. Unlike ALC-0315 and SM-102, DMKD-based LNPs, which use a cationic rather than an ionizable lipid, have a bilayer liposome structure. The inclusion of negatively charged nucleic acids either inside or on the particle surface stabilizes this structure. Despite a mere 2 mol% difference in PS composition, DMKD shows more instability and size increase upon mRNA encapsulation compared to DMKD-PS. PS appears to function as a helper lipid in the LNP structure, providing fluidity and contributing to a more stable DMKD formulation [35]. Transmission electron microscopy (TEM) images of DMKD-PS supported its spherical and bilayer shape, with a diameter of approximately 200 nm.

### 2.4. mRNA Protection Analyses

**Figure 3:**
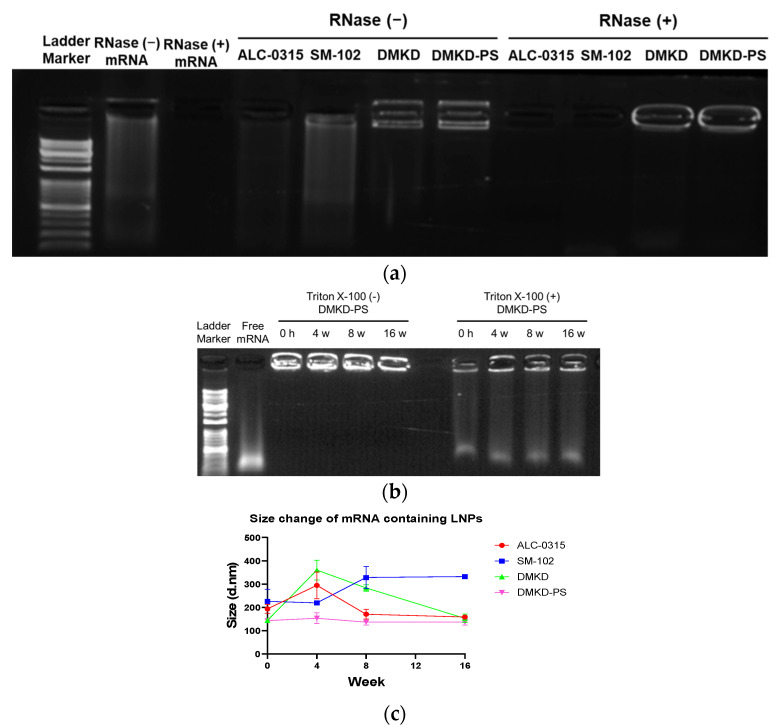
The mRNA protection by DMKD-PS LNPs. (**a**) LNPs encapsulating mRNA were treated with 20 µg of RNase A and incubated at 37 °C overnight, followed by analyses on a 1% agarose gel to assess mRNA integrity. (**b**) To evaluate the structural integrity of mRNA within the LNPs, electrophoresis was conducted after refrigerating the samples for up to 16 weeks. (**c**) The stability of mRNA-loaded LNPs was monitored for 4, 8, and 16 weeks (*n* = 3). Error bars represent the mean ± standard deviation (S.D.) of three independent experiments.

mRNA protection analyses were conducted to determine the storage conditions under which various LNPs could effectively protect mRNA from RNase A. The encapsulation of mRNA by the LNPs was verified using a gel retardation assay (Figure 3a). In the absence of RNase A treatment, mRNA was detected in the LNPs. However, the ALC-0315 and SM-102 LNPs displayed smeared bands, indicating suboptimal mRNA binding. In contrast, no mRNA was released from the DMKD and DMKD-PS LNPs. Upon RNase A treatment, mRNA was not detected in the ALC-0315 and SM-102 LNPs, suggesting inadequate protection, whereas DMKD and DMKD-PS LNPs showed robust mRNA binding. The primary aim of this gel retardation assay is to evaluate the strength of the binding between LNPs and mRNA, particularly their ability to retain mRNA without external release. In the assay, LNPs that are strongly bound to mRNA do not move through the agarose gel pores, remaining in the well. While LNPs with ALC-0315 and SM-102 partially retained mRNA in the well, most were unable to capture nucleic acids effectively and migrated through the gel. In contrast, DMKD-based LNPs exhibited a strong affinity for nucleic acids, mostly remaining in the well. When exposed to RNase A, only the DMKD-based LNPs retained nucleic acids, indicating superior protection of mRNA from RNase and a strong, lasting bond that prevents mRNA release into the external environment. 

Furthermore, the structural integrity of mRNA with DMKD-PS LNPs at 4C was assessed using a gel retardation assay (Figure 3b). Remarkably, the DMKD-PS LNPs maintained mRNA complexation even after 16 weeks. Disruption of DMKD-LNPs using Triton X-100 resulted in smeared bands, indicating that the mRNA was securely entrapped within the particles, which maintained its structural integrity under storage conditions. 

Additionally, the stability of LNPs carrying mRNA was tested by refrigerating and monitoring the changes in particle size for 16 weeks (Figure 3c). Despite the extended refrigeration period, the DMKD-PS LNPs demonstrated minimal size changes, unlike other particles that exhibited significant size alterations. Observing the size of mRNA-captured LNPs at refrigeration temperature over 16 weeks, DMKD-PS shows consistent size, indicating structural stability. This outcome arises from the strong binding between mRNA and LNPs, which maintains their structural integrity over time. Notably, factors such as particle size and stability are influenced by the binding ratio of nucleic acids to lipid carriers [36], underscoring the importance of this interaction in determining the physical properties of the LNPs. These results demonstrate that DMKD-PS LNPs not only effectively protect mRNA from RNase A degradation but also maintain their stable structures and mRNA protection capability, even after prolonged refrigeration.

### 2.5. Inherent Cytotoxicity of DMKD-PS

**Figure 4:**
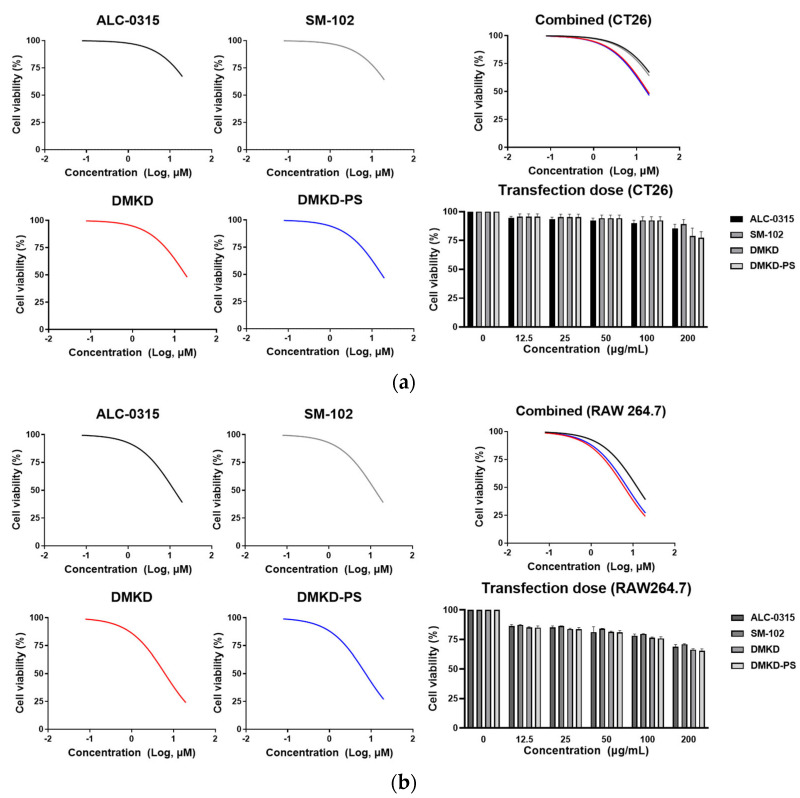
The in vitro cytotoxicity analyses of DMKD-PS LNPs. (**a**) CT26 and (**b**) RAW 264.7 cells were exposed to various LNPs, including ALC-0315, SM-102, DMKD, and DMKD-PS, for 24 h. Subsequently, the viability of the treated cells was quantified by measuring the absorbance at 450 nm (*n* = 6). Error bars denote the mean ± standard deviation (S.D.) derived from three independent experiments.

To assess the cytotoxicity of the prepared LNPs, CT26 and RAW 264.7 cells were treated with various concentrations of the prepared LNPs, using a serial dilution from 20 μM down to 0.15 μM. The cell viability was measured at a wavelength of 450 nm after 24 h using a spectrophotometer (Figure 4a,b). The results indicated that DMKD and DMKD-PS LNPs exhibited relatively higher cytotoxicity than the ALC-0315 and SM-102 LNPs in both cell types. The ALC-0315 and SM-102 LNPs displayed similar viability in both cell types. Although the DMKD and DMKD-PS LNPs showed comparable viability in CT26 cells, the DMKD LNPs appeared to be slightly less toxic to RAW 264.7 cells.

In addition, a cytotoxicity test was conducted to estimate the adequate LNP concentration based on the FDA-approved dosage for mRNA vaccine delivery. Concentrations up to 12.5 μg/mL were tested, following a serial dilution from the maximum permissible concentration of 200 μg/mL [37]. In CT26 cells, all LNPs exhibited negligible toxicity up to 100 μg/mL, but at 200 μg/mL, the DMKD and DMKD-PS LNPs showed slightly reduced cell viability by approximately 75%. 

In RAW 264.7 cells, cell viability was above 80% at 12.5 ug/mL and the viability was maintained at >75%, even at 200 μg/mL for all LNPs. Although the DMKD and DMKD-PS LNPs displayed a slightly higher toxicity than the ALC-0315 and SM-102 LNPs at concentrations up to 20 μM, they showed comparable cell viability to each other at the FDA-guided concentration of 200 μg/mL.

The higher toxicity of DMKD-based LNPs compared to other types is likely attributable to the positive charge of DMKD, as cytotoxicity is known to be induced by cationicity [38,39]. However, given that no significant difference in toxicity was noted at a concentration of 200 μg/mL, determining an appropriate dosage is crucial for minimizing cytotoxicity.

### 2.6. In-Vitro GFP mRNA Transfection of DMKD-PS

**Figure 5:**
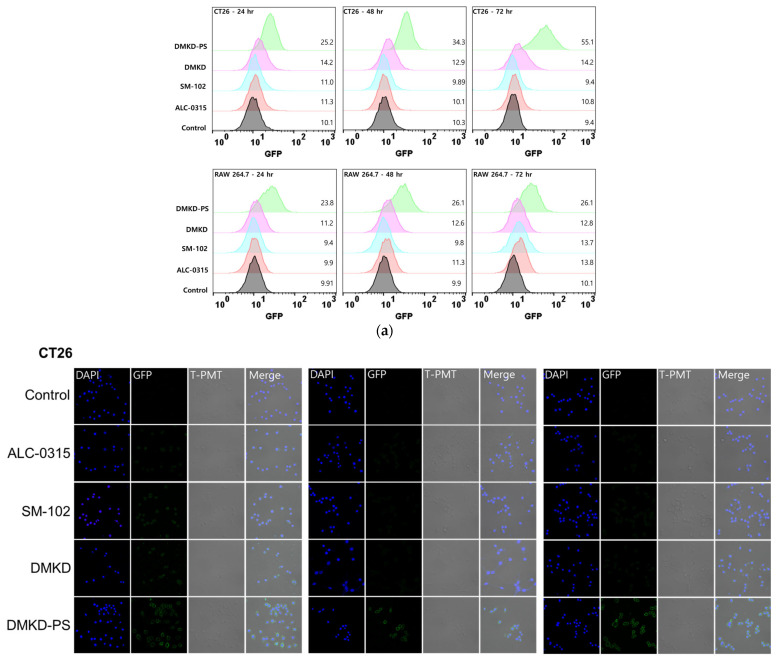

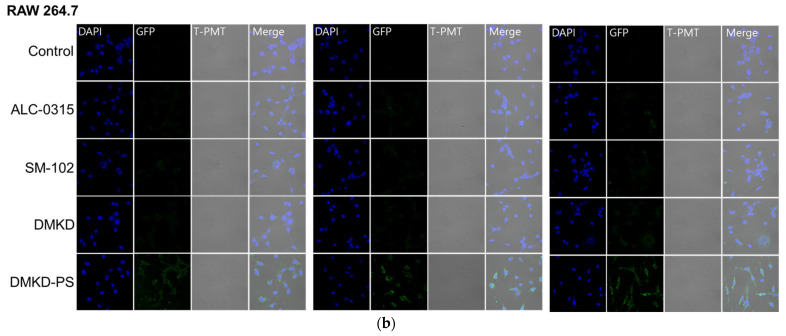
GFP mRNA transfection by DMKD-PS LNPs to CT26 and RAW 264.7 cells. (**a**) Flow cytometry analyses of CT26 and RAW 264.7 cells transfected with GFP mRNA complexed with ALC-0315, SM-102, DMKD, or DMKD-PS LNPs, conducted 24, 48, and 72 h after transfection. (**b**) Confocal microscopy images of both cell lines after transfection with the GFP-mRNA-LNP complexes for 24, 48, and 72 h. Magnification; ×200.

The fluorescence intensity of GFP was measured in CT26 and RAW 264.7 cells using flow cytometry, following transfection with GFP mRNA loaded in various LNPs (Figure 5a). The flow cytometry results indicated a greater shift in the histogram for DMKD-PS in CT26 cells than for other LNPs, implying higher GFP expression. The numbers indicated on the right side of each histogram represent the geometric mean value of GFP fluorescence. Notably, GFP fluorescence in CT26 cells progressively increased with transfection time. In contrast, the other LNPs exhibited smaller histogram shifts. In RAW 264.7, DMKD-PS also exhibited the strongest GFP fluorescence intensity, but the intensity remained consistent across all time points. Thus, both transfected CT26 and RAW 264.7 cells demonstrated the superior mRNA translation efficiency of DMKD-PS LNPs compared to the other LNPs. This observation was further corroborated by visual confirmation by confocal microscopy (Figure 5b), which mirrored the flow cytometry findings.

RAW 264.7 cells, unlike CT26 cells, exhibit a balance between the rate of mRNA translation and protein degradation by proteasomes. This characteristic, stemming from the murine macrophage cell line RAW 264.7, contributes to their efficient antigen degradation capability. As a result, achieving consistent expression of specific proteins in RAW 264.7 is challenging because of their potent antigen-degrading ability [37,40].

### 2.7. In-Vivo Biodistribution

**Figure 6:**
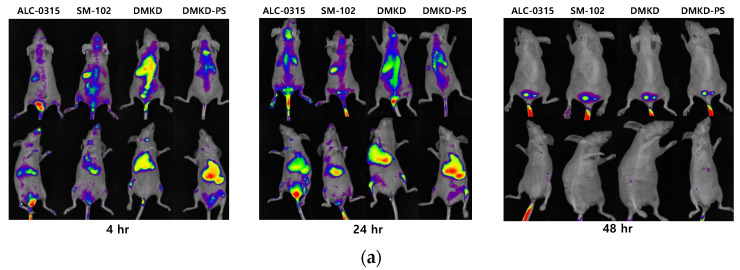

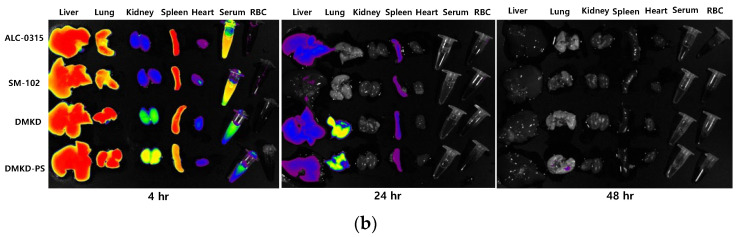
In vivo and ex vivo biodistribution analyses of various LNPs. (**a**) BALB/c nude mice were intravenously injected with DiD-labeled LNPs, followed by whole-body imaging 4, 24, and 48 h post injection. (**b**) After whole-body imaging, the major organs were dissected, and their ex vivo fluorescence signals were imaged.

To investigate their in vivo distribution and clearance from the body, fluorescence-labeled LNPs were intravenously administered to mice. The distribution of the nanoparticles was monitored 4, 24, and 48 h after administration using a fluorescence imaging system (Figure 6a). The initial observations indicated a uniform distribution of all LNPs across the body surface. However, ex vivo analyses at the 4 and 24 h marks showed that the nanoparticles predominantly localized within the liver and lungs (Figure 6b). LNPs containing DMKD demonstrated greater accumulation in the kidneys than those containing ALC-0315 and SM-102, with a concurrent reduction in serum fluorescence.

Relative to other lipid nanoparticles, DMKD-based nanoparticles exhibited stronger fluorescence signals in the lungs and liver, whereas the spleen and serum showed relatively lower signals. This distribution pattern is considered to arise from the positive surface charge of nanoparticles, leading to enhanced entrapment in the reticuloendothelial system (RES) [41,42,43]. However, all nanoparticles, including DMKD-PS, were cleared from the body within 48 h. Thus, the in vivo distribution of DMKD-based nanoparticles, as well as others, was found to be disassembled, metabolized, and eliminated from the body within the same timeframe.

## 3. Materials and Methods

### 3.1. Materials

1,2-distearoyl-sn-glycero-3-phosphoethanolamine-N-[methoxy(polyethylene glycol)-2000] (DSPE-PEG2000-amine), cholesterol, 1,2-distearoyl-sn-glycero-3- phosphotidylcholine (DSPC), and 1,2-dimyristoyl-rac-glycero-3-ethoxypolyethylene glycol-2000 (PEG-DMG) were purchased from Avanti Polar Lipid, Inc. (Alabaster, AL, USA). DMKD cationic lipid was obtained from the Biomedical Laboratory Science, Yonsei University MIRAE Campus (Wonju, Republic of Korea). 2-hexyl-decanoic acid, 1,1′-[[(4-hydroxy butyl)imino]di-6,1-hexanediyl] ester (ALC-0315), 8-[(2-hydroxyethyl) [6-oxo-6-(undecyloxy) hexyl]amino]-octanoic acid, 1-octylnonyl ester (SM-102), and α-[2-(ditetradecylamino)-2-oxoethyl]-ω-methoxy-poly(oxy-1,2-ethanediyl) (ALC-0159) were purchased from Cayman Chemical (Ann Arbor, MI, USA). Cellomax^TM^ for cell viability was supplied by Precaregene (Uiwang, Republic of Korea). Restriction enzyme (*Eco*RI, *Eco*RV, *Not*I, and *Spe*I), competent cell (*E. coli* DH5α), and EZ™ T7 High-Yield In Vitro Transcription Kit were supplied by Enzynomics (Daejeon, Republic of Korea). pEGFP-N1 was purchased from addgene, and pIVTRup was a gift from Ángel Raya (Addgene plasmid #101362; http://n2t.net/addgene:101362, accessed on 9 July 2020; RRID:Addgene_101362). Ambion™ Cap Analog [m7G(5′)ppp(5′)G] was purchased from Thermo Fisher Scientific (Waltham, MA, USA). Dokdo Plasmid Mini-Prep Kits were purchased from ELPIS Biotech (Daejeon, Republic of Korea).

### 3.2. Cell Lines and Cell Culture

Murine macrophages (RAW 264.7, ATCC TIB-71^TM^) and murine colorectal carcinoma cells (CT26, ATCC CRL-2638^TM^) were purchased from American Type Culture Collection (Manassas, VA, USA). RAW 264.7 and CT26 cells were cultured in Dulbecco’s modified Eagle Medium (DMEM; BYLABS, Hanam, Republic of Korea) supplemented with 10% EqualFETAL^®^ bovine serum (Atlas biologicals, Fort Collins, CO, USA), 100 IU/mL penicillin, 100 μg/mL streptomycin, and 0.25 μg/mL amphotericin B. All cell lines were incubated at 37 °C in a humidified 5% CO_2_ incubator. The cells were subcultured to 80–90% confluency.

### 3.3. Synthesis of Enhanced Green Fluorescence Protein mRNA

The pEGFP-N1 plasmid inserted *E. coli* DH5α were enriched in LB containing kanamycin overnight at a 100 μg/mL concentration. Subsequently, the pEGFP-N1 plasmid was extracted using a mini-prep method. A restriction enzyme digestion was performed at 37 °C overnight using *EcoR*I and *Not*I on approximately 10 μg of pEGFP-N1, and 1% agarose gel electrophoresis was conducted to separate DNA fragments corresponding to enhanced green fluorescence protein (EGFP). After electrophoresis, gel extraction was performed to extract EGFP sequences. The pIVTRup was digested overnight by *Eco*RV at 37 °C, and then calf intestine alkaline phosphatase (CIAP) was treated to prevent self-ligation of the plasmid. The extracted EGFP sequence and digested pIVTRup were mixed in a 1:10 weight ratio, and DNA ligation was performed at a refrigerated temperature overnight using T4 DNA ligase. The final recombinant plasmid was confirmed using 1% agarose gel electrophoresis and DNA sequencing.

Before initiating in vitro transcription (IVT), pDNA was linearized using *Spe*I restriction enzyme to minimize the production of undesired IVT products. IVT was performed with 2 μg of the pIVTRup-EGFP template and the T7 IVT kit according to the manufacturer’s protocol, and 5′ capping was performed simultaneously by adding m7G cap analog at a concentration of 4 times the reacting GTP. After the reaction, mRNA was added to two volumes of ethanol and incubated at −20 °C for 30 min. Next, centrifugation was performed at 14,000× *g* for 30 min. The supernatant was removed, and the pellet was resuspended in 1 mL of RNase-free water. The synthesized mRNA was stored at −80 °C. 

### 3.4. Preparation of DMKD-PS

An optimized ratio of DMKD, DSPE-PEG_2000_-amine, cholesterol, and PS (mol %) was dried under nitrogen gas and evaporated under vacuum for 1 h. After evaporation, the lipid film was hydrated with 1 mL normal saline solution. DMKD-PS was sonicated three times for 15 min each at 10 min intervals in a glass tube using a bath-type sonicator (Elma Schmidbauer GmbH, Singen, Germany) at a frequency of 37 kHz and power of 60 W. The resulting suspension was extruded ten times through a polycarbonate membrane with a pore size of 100 nm using an extruder (Avanti Polar Lipids, Alabaster, AL, USA). For comparison, other types of LNPs were prepared in the same manner as DMKD-PS using other ionizable cationic lipids, ALC-0315 and SM-102. In addition, DMKD LNPs, from which only PS was removed, were prepared. The compositions of the prepared LNPs are listed in Table S1.

The lipid film method was used to prepare LNPs for mRNA delivery. Initially, mRNA was produced via in vitro transcription. Subsequently, the mRNA levels were 1:1 to 10:1. The N/P ratio refers to the ratio between the nitrogen atoms in the lipid component and the phosphate groups in the nucleic acids, reflecting their binding interactions. The mixture was vortexed for 30 s and incubated at room temperature for 30 min to facilitate mRNA complexation with the LNPs. The success of this binding process was evaluated by electrophoresis on a 1% agarose gel, followed by visualization under UV illumination. Finally, the sizes and surface charges of the prepared LNPs (ALC-0315, SM-102, DMKD, and DMKD-PS) were measured thrice by the dynamic light scattering (DLS) method using a Zetasizer Nano-ZS90 (Malvern Instruments Ltd., Malvern, UK). The data were statistically analyzed using GraphPad Prism 9 software (GraphPad Software, Inc., Boston, MA, USA).

### 3.5. Transmission Electron Microscopy Analysis

The morphologies of ALC-0315, SM-102, DMKD, and DMKD-PS were observed by transmission electron microscopy (TEM) (JEM-F200; JEOL Ltd., Tokyo, Japan). The solutions containing 100 μg of particles were loaded on carbonyl-coated 400-mesh copper grids coated with carbon for 15 min. For negative staining, 10 μL of UranyLess EM stain solution (Electron Microscopy Sciences, Harfield, UK) was placed on the grid for 10 min, then removed by Whatman^TM^ filter paper (Cytiva, Marlborough, MA, USA), and dried for 10 min at room temperature. TEM images were acquired at an acceleration voltage of 80 kV.

### 3.6. mRNA Protection Analysis

To evaluate the protective ability of the lipid nanoparticles against RNase, GFP mRNA was incorporated into each lipid nanoparticle. After mixing with 20 µg of RNase A and allowing the reaction to proceed overnight at room temperature, the samples were subjected to electrophoresis on a 1% agarose gel for 15 min at 100 V. To further assess the mRNA encapsulation and protection capabilities of DMKD-PS, the samples were stored at 4C for durations of 4, 8, and 16 weeks. Subsequently, the particles were intentionally disrupted using Triton-X 100, a nonionic detergent, and electrophoresis was performed on agarose gel for 15 min at 100 V.

### 3.7. Cytotoxicity Assay

The cytotoxicity of DMKD-PS was examined by Cellomax^TM^ cell viability assay kit (Precaregene, Uiwang, Republic of Korea). CT26 and RAW 264.7, at a density of 1×10^4^ cells/well, were cultured for 24 h. The cells were treated with various concentrations of LNPs and incubated for 24 h and 48 h. Then, 10 μL of CelloMax^TM^ solution was added to each well, which was further incubated for 2 h. After incubation, absorbance was measured at 450 nm using a microplate reader (TECAN Group, Männedorf, Switzerland).

### 3.8. In Vitro GFP mRNA Transfection

#### 3.8.1. Flow Cytometry Analysis

To compare the mRNA transfection efficiency of the prepared LNPs (ALC-0315, SM-102, DMKD, DMKD-PS) containing 2 g of GFP, mRNA was added to CT26 and RAW 264.7 cells in 6-well plate (2 × 10^5^ cells/well) and then cultured for 24, 48, and 72 h. After being trypsinized and washed with 1× PBS, the cells were treated with 2% paraformaldehyde for 5 min at 4 °C. Transfected cells were analyzed using FACSCalibur (Becton Dickinson, San Jose, CA, USA).

#### 3.8.2. Confocal Microscopy Analysis

CT26 and RAW 264.7, at a density of 1 × 10^5^ cells per well, were placed onto cover slips in 6-well plates and allowed to grow for 24 h. Different types of LNPs containing 2 g of GFP mRNA were then added to cells. The cells with LNPs were incubated for varying durations for 24, 48, and 72 h at 37 °C in a medium without serum. Following each treatment period, the cells were washed twice with a cold 1 × PBS solution at pH 7.4 and then fixed using a 2% paraformaldehyde solution at 4 °C for 10 min in a dark environment. After fixation, the cells were washed thrice with 1 × PBS. They were then stained with DAPI (4′,6-diamidino-2-phenylindole) solution for 10 min in the dark. The stained cells were mounted on slides for observation. The slides were examined under a confocal laser scanning microscope (LSM 710, Carl Zeiss, Heidenheim, Germany).

### 3.9. In Vivo Biodistribution Analysis

To formulate LNPs (ALC-0315, SM-102, DMKD, and DMKD-PS), the lipid mixtures were evaporated under a stream of nitrogen gas, followed by vacuum desiccation for 1 h. Subsequently, the LNPs were sonicated and extruded using a 100 nm polycarbonate membrane filter following a previously described method. To visualize the LNPs, DiD (1,1′-dioctadecyl-3,3,3′,3′-tetramethylindodicarbocyanine, 4-chlorobenzenesulfonate salt), a type of lipophilic dye, was used for staining. Each LNP preparation involved 5 mg of lipids and 100 μg of DiD. Excess or unbound DiD was removed using a PD-10 desalting column (GE Healthcare, Little Chalfont, USA). The mice were administered 200 μL of the prepared LNPs through tail vein injection (1 mg of total lipids). After injection, fluorescence images of the mice were obtained at 4, 24, and 48 h using an FOBI in vivo imaging system (CELLGENTEK, Daejeon, Republic of Korea). After capturing whole-body fluorescence images, the mice were euthanized, and the major organs were extracted for further examination. The fluorescence intensities of all dissected organs were quantified using the NEO image software version 3.3 (CELLGENTEK, Daejeon, Republic of Korea).

## 4. Conclusions

The novel contribution of this study primarily lies in the enhanced stability and efficiency of the newly developed DMKD-PS lipid nanoparticles (LNPs) for mRNA delivery. Unlike previously used lipid nanoparticles, which require storage at temperatures below minus 80 degrees Celsius, the DMKD-PS LNPs demonstrated remarkable structural stability, maintaining size consistency and effectively protecting mRNA even under prolonged refrigeration. This represents a significant advancement in terms of practical storage and handling of mRNA carriers. Additionally, the DMKD-PS LNPs exhibited remarkable resilience in the presence of RNase A, safeguarding the mRNA effectively. Furthermore, even when challenged with Triton X-100, a potent detergent, these nanoparticles resisted complete destruction, underscoring their enhanced stability compared to existing lipid nanoparticles. An important breakthrough of our study is the cytotoxicity profile of DMKD-PS. Despite being strongly positively charged, these LNPs exhibited cytotoxicity levels comparable to neutral-charged lipid nanoparticles, a notable achievement in the field. Moreover, the efficient delivery of GFP mRNA to cells suggests that lower dosages of DMKD-PS can achieve high transmission efficiency, potentially reducing toxicity. Lastly, the systemic administration of DMKD-PS nanoparticles showed that they undergo normal metabolic processing and are safely expelled from the body. While similar to conventionally available mRNA carriers in certain aspects, DMKD-PS’s unique characteristics and performance indicate its potential applicability in various fields, including mRNA-based cancer vaccines, warranting further research and exploration. In summary, the DMKD-PS lipid nanoparticles mark a significant step forward in mRNA delivery technology, combining enhanced stability, efficient mRNA protection, low cytotoxicity, and effective systemic processing, thereby opening new avenues in mRNA therapeutic applications.

## 5. Patents

Park, Y. S., Choi, K. C., & Lee, D. H. (2022). Cationic lipid nanoparticles as mRNA vaccines (Korean Patent No. 10-2022-0044633). Korean intellectual property offices.

## Figures and Tables

**Table 1:** Physicochemical properties of the various LNPs.

	mRNA	Size ^(a,b)^ (nm)	Polydispersity Index (PDI)	ζ-Potential (mV)
ALC-0315	-	193.4 ± 15.6	0.424 ± 0.054	−1.2 ± 1.3
	+	143.7 ± 3.5 (▼)	0.290 ± 0.025 (▼)	−4.3 ± 0.5 (▼)
SM-102	-	226.0 ± 41.9	0.497 ± 0.011	5.6 ± 0.2
	+	158.6 ± 5.4 (▼)	0.415 ± 0.021 (▼)	−8.1 ± 0.7 (▼)
DMKD	-	144.5 ± 3.0	0.325 ± 0.010	29.9 ± 2.3
	+	206.7 ± 3.5 (▲)	0.367 ± 0.012(▲)	27.7 ± 1.3 (▼)
DMKD-PS	-	134.1 ± 1.0	0.316 ± 0.026	42.8 ± 1.0
	+	150.7 ± 8.6 (▲)	0.223 ± 0.006 (▼)	26.1 ± 0.8 (▼)

^(a)^ The Diameter, PDI, and Zeta potential were measured three times using a particle analyzer. ^(b)^ Average diameter ± S.D.; PDI ± S.D.; average ζ-potential ± S.D.

## Data Availability

Data are contained within the article.

## Supplementary Materials

The following supporting information can be downloaded from: https://www.mdpi.com/article/10.3390/ijms25031388/s1.
