# Supplementary material for: Novel Lipid Nanoparticles Stable and Efficient for mRNA Transfection to Antigen-Presenting Cells
Source: Int J Mol Sci. 2024 Jan 23;25(3):1388. doi: 10.3390/ijms25031388 (PMC10855810; doi:10.3390/ijms25031388)
Supplement: Supplementary file 1 [file ijms-25-01388-s001.zip › ijms-2832183-supplementary.pdf]

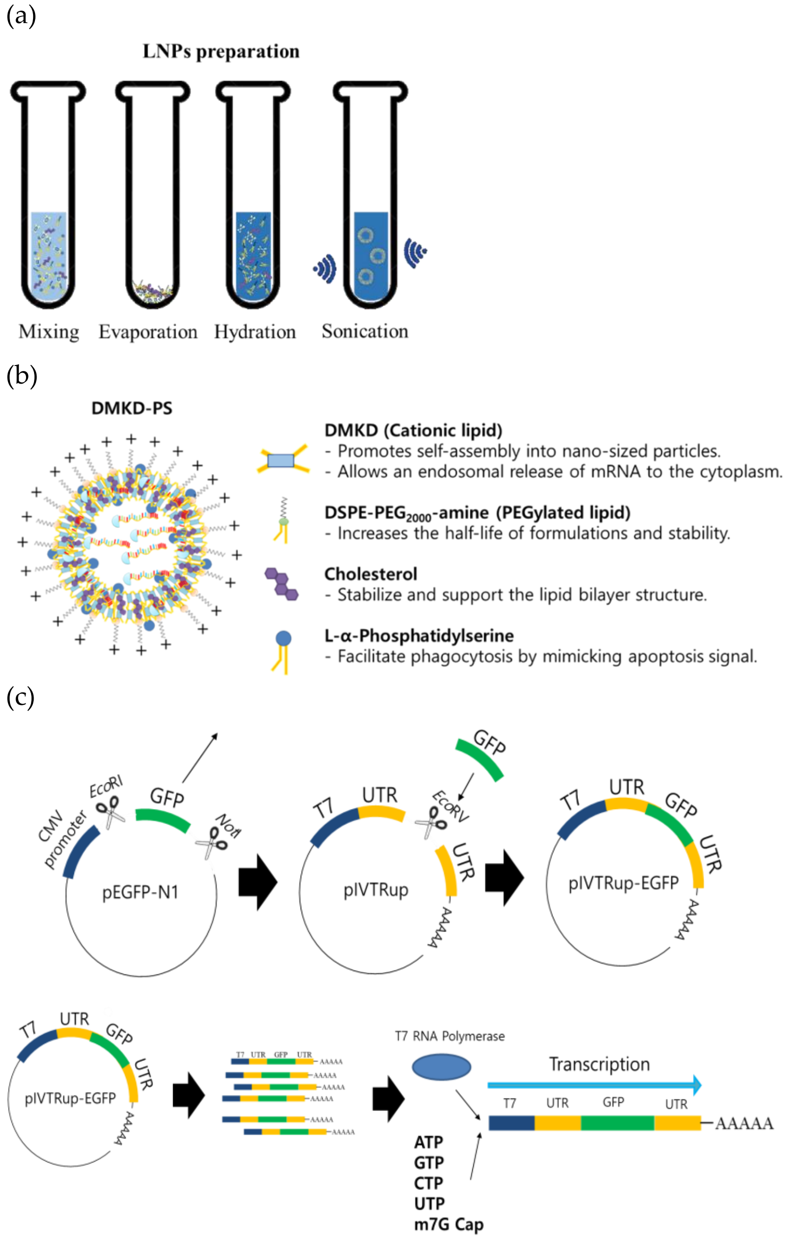

Figure S1. Schematic illustration of DMKD-PS with mRNA. (a) The DMKD-PS LNPs were prepared by the lipid film method and (b) each component constituting the DMKD-PS LNPs. (c) The mRNA was transcribed *in vitro* followed by mRNA complexation.

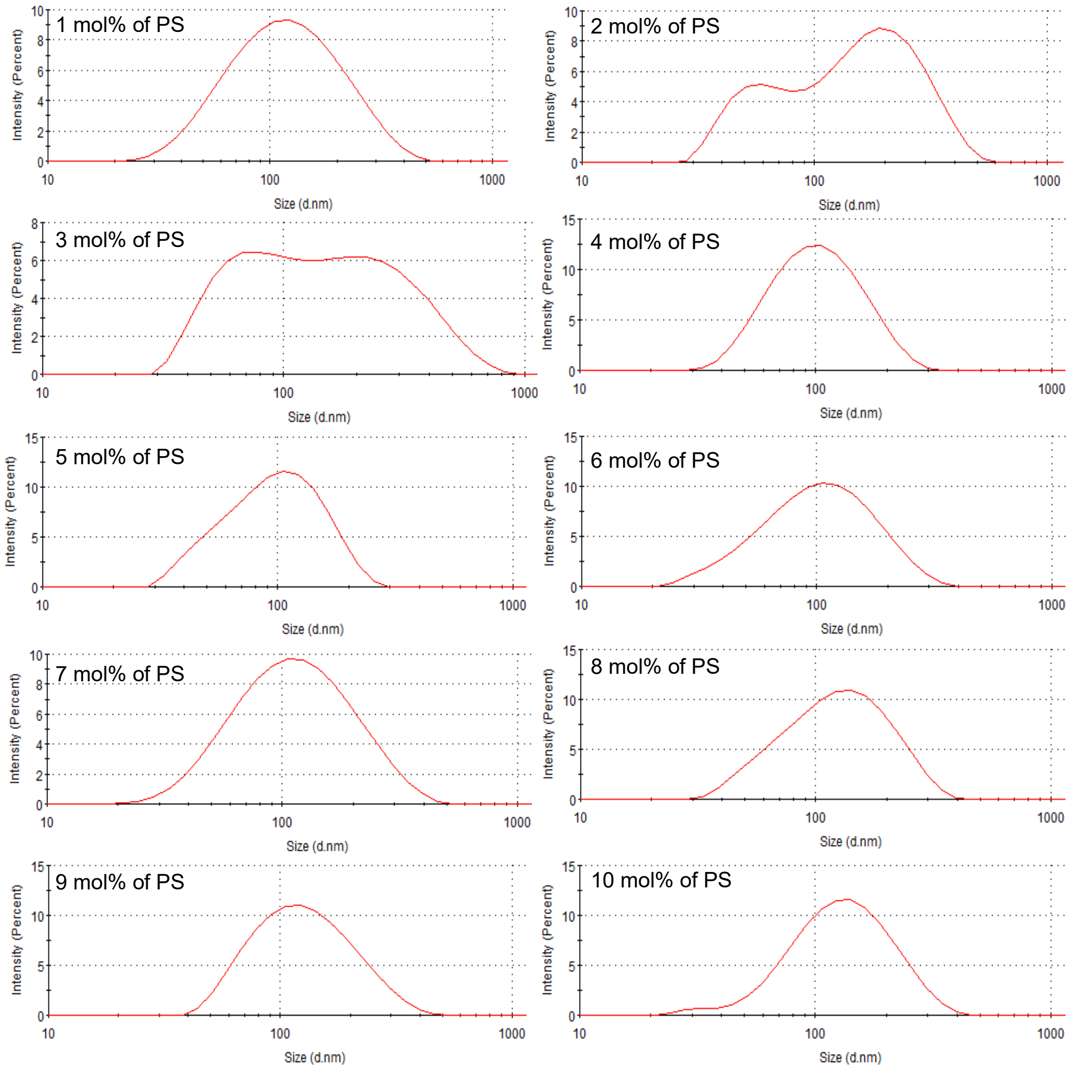

Figure S2. The size distribution of DMKD-PS containing the various molar ratios of PS. DMKD-PS LNPs were produced in 10 unique compositions by altering the PS molar ratio while keeping the DMKD, cholesterol, and DSPE-PEG2000 proportions consistent and then subjected to DLS analyses.

(a) N/P ratio = 1:1

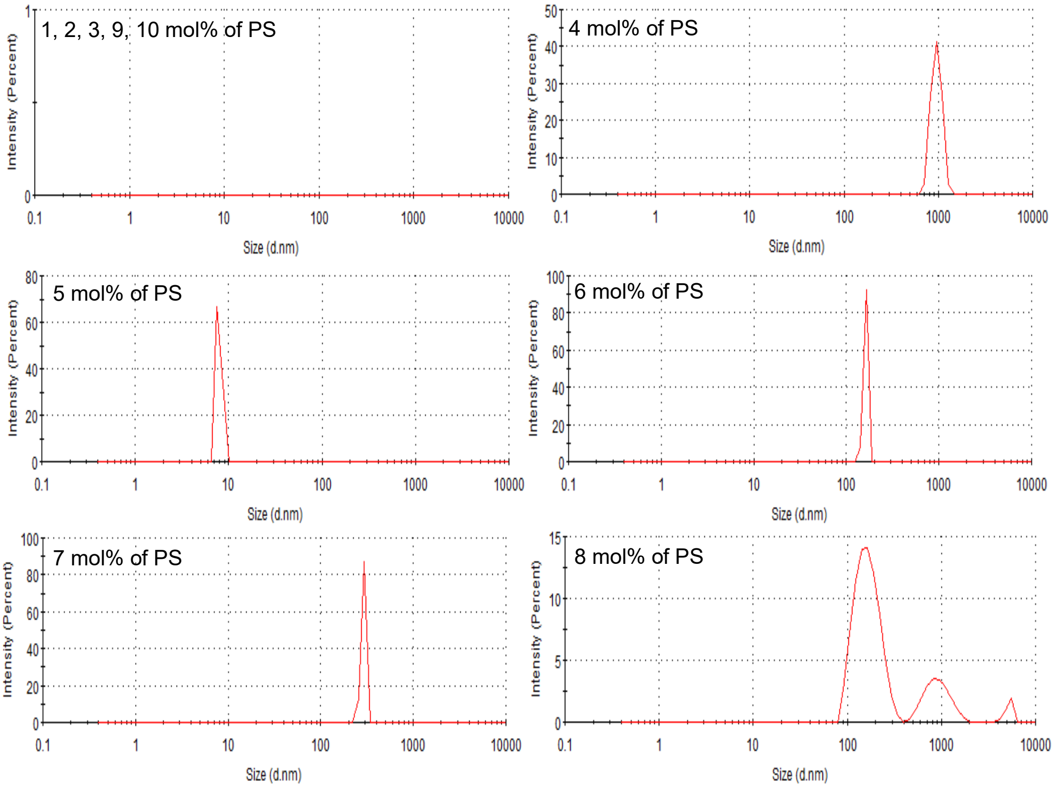

1, 2, 3, 9, 10 mol% of PS
4 mol% of PS
5 mol% of PS
6 mol% of PS
7 mol% of PS
8 mol% of PS
Intensity (Percent)
Size (d.nm)
0.1
1
10
100
1000
10000

(b) N/P ratio = 2:1

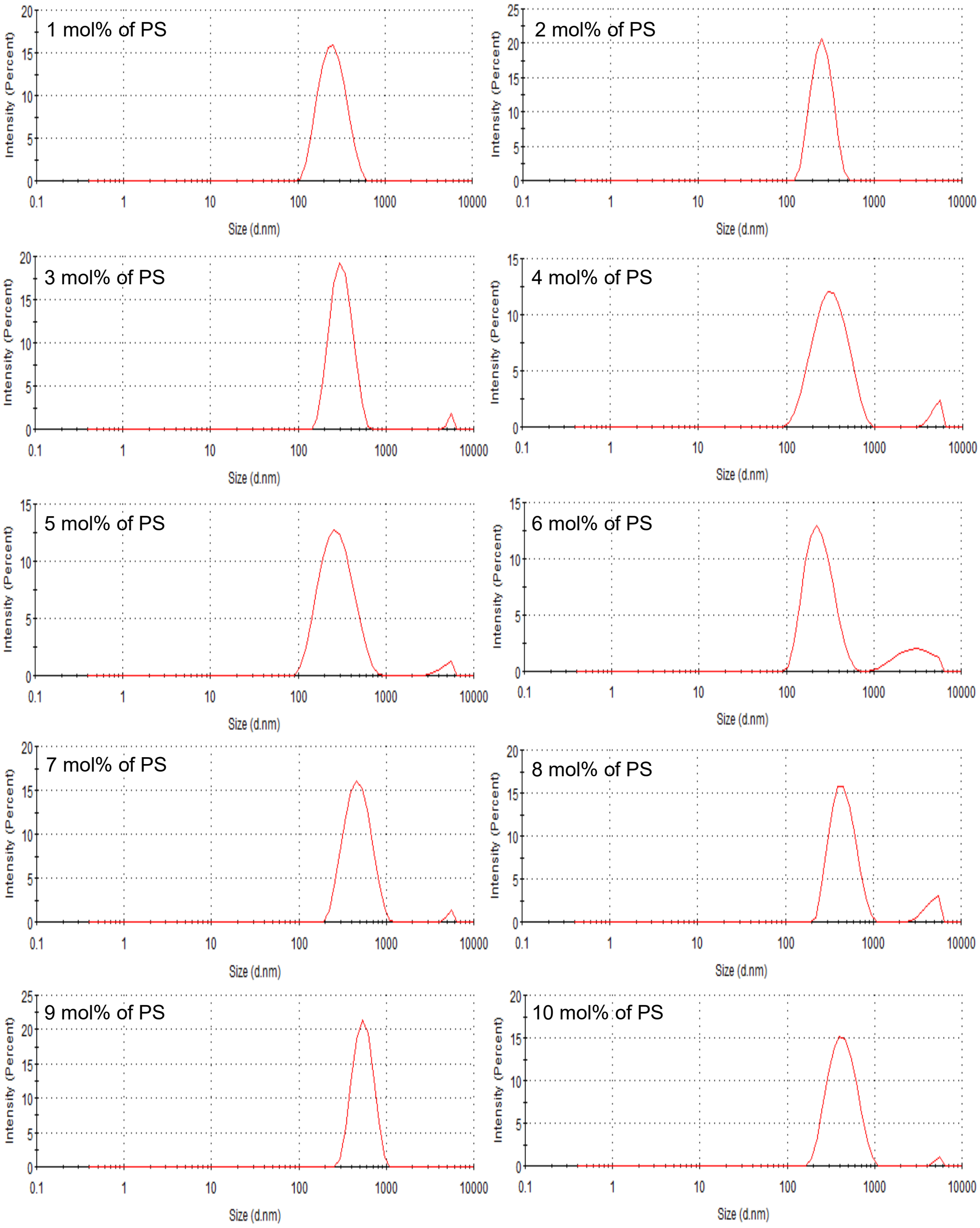
1 mol% of PS
2 mol% of PS
3 mol% of PS
4 mol% of PS
5 mol% of PS
6 mol% of PS
7 mol% of PS
8 mol% of PS
9 mol% of PS
10 mol% of PS
Intensity (Percent)
Size (d.nm)
0.1
1
10
100
1000
10000

(c) N/P ratio = 3:1

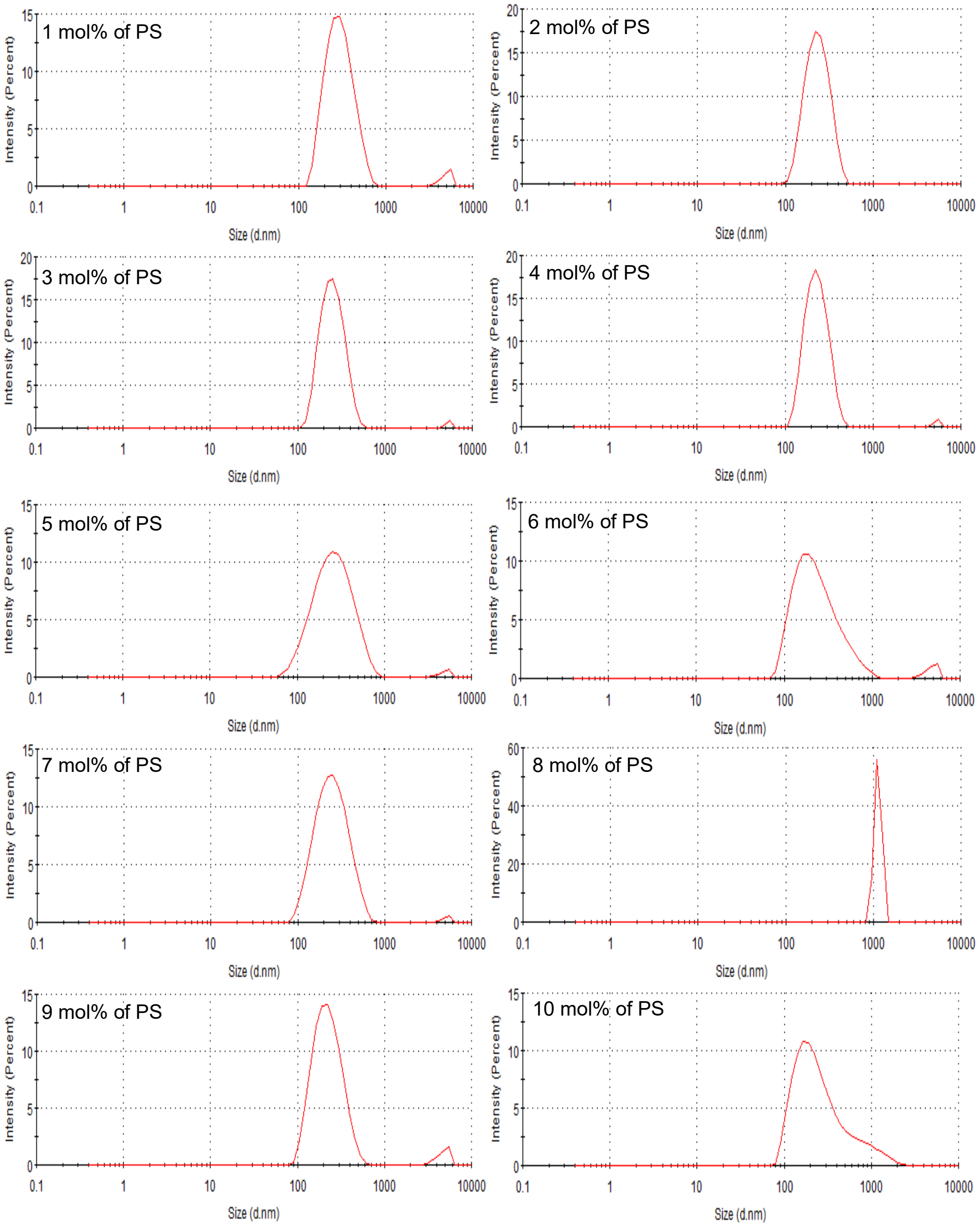
1 mol% of PS
2 mol% of PS
3 mol% of PS
4 mol% of PS
5 mol% of PS
6 mol% of PS
7 mol% of PS
8 mol% of PS
9 mol% of PS
10 mol% of PS
Intensity (Percent)
Size (d.nm)
0.1
1
10
100
1000
10000

(d) N/P ratio = 4:1

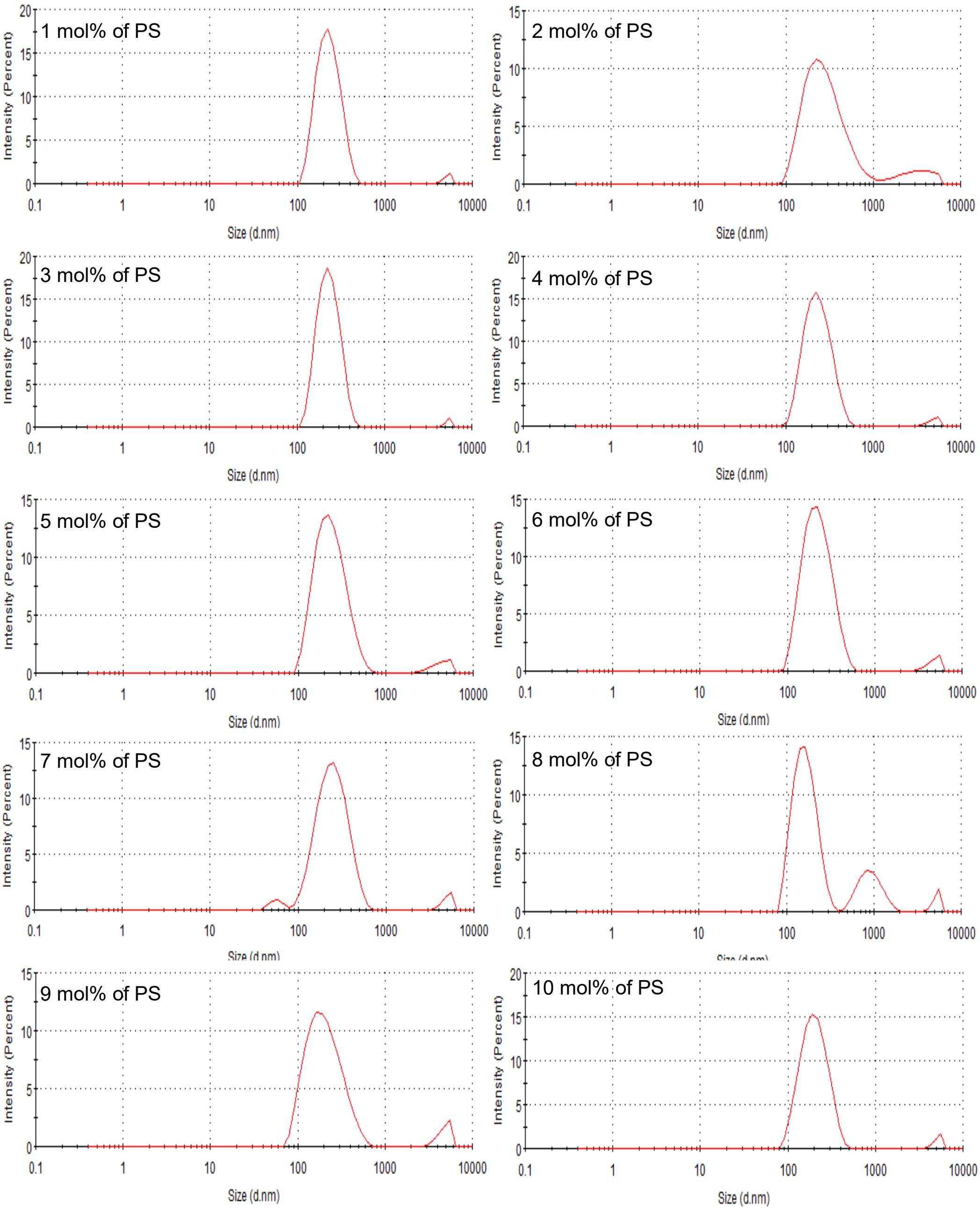

Figure S3. The size distribution of DMKD-PS containing varied mole% of PS after mRNA complexation at various N/P ratios. The LNPs were prepared with varied mole% of PA and then complexed with GFP mRNA at the ratios from (a) 1:1, (b) 2:1, (c) 3:1, and (d) 4:1.

Table S1. The components of various LNPs used in this study

LNPs	Components	Mol%
**ALC-0315**	ALC-0315, Cholesterol, DSPC, ALC-0159	46.3 : 42.7 : 9.4 : 1.6
**SM-102**	SM-102, Cholesterol, DSPC, PEG-DMG	50 : 38.5 : 10 : 1.5
**DMKD**	DMKD, Cholesterol, DSPE-PEG	55 : 25 : 20
**DMKD-PS**	DMKD, Cholesterol, DSPE-PEG, PS	55 : 25 : 18 : 2
